# Clinical features of intussusception in children secondary to small bowel tumours: a retrospective study of 31 cases

**DOI:** 10.1186/s12887-024-04717-y

**Published:** 2024-04-01

**Authors:** Li Wang, Hanwen Zhang, Dayong Wang, Qiulong Shen, Liuming Huang, Tingting Liu

**Affiliations:** 1 Department of Emergency Surgery, Beijing Children’s Hospital, Capital Medical University, National Center for Children’s Health, Beijing, 100045 China; 2 Present Address: Beijing Children’s Hospital, Capital Medical University, National Center for Children’s Health, No.56, Nanlishi Road, Beijing, 100045 PR China

**Keywords:** Small bowel tumours, Intussusception, Children, Surgery, Secondary

## Abstract

**Background:**

Summarizing the clinical features of children with intussusception secondary to small bowel tumours and enhancing awareness of the disease.

**Methods:**

Retrospective summary of children with intussusception admitted to our emergency department from January 2016 to January 2022, who underwent surgery and were diagnosed with small bowel tumours. Summarize the types of tumours, clinical presentation, treatment, and prognosis.

**Results:**

Thirty-one patients were included in our study, 24 males and 7 females, with an age of onset ranging from 1 m to 11y 5 m. Post-operative pathology revealed 4 types of small intestinal tumour, 17 lymphomas, 10 adenomas, 4 inflammatory myofibroblastomas and 1 lipoma. The majority of tumours in the small bowel occur in the ileum (83.9%, 26/31). Abdominal pain, vomiting and bloody stools were the most common clinical signs. Operative findings indicated that the small bowel (54.8%, 17/31) and ileocolic gut were the main sites of intussusception. Two types of procedure were applied: segmental bowel resection (28 cases) and wedge resection of mass in bowel wall (3 cases). All patients recovered well postoperatively, with no surgical complications observed. However, the primary diseases leading to intussusception showed slight differences in long-term prognosis due to variations in tumor types.

**Conclusions:**

Lymphoma is the most common cause of intussusception in pediatric patients with small bowel tumours, followed by adenoma. Small bowel tumours in children tend to occur in the ileum. Therefore, the treatment of SBT patients not only requires surgeons to address symptoms through surgery and obtain tissue samples but also relies heavily on the expertise of pathologists for accurate diagnosis. This has a significant impact on the overall prognosis of these patients.

## Background

Intussusception in children is a common clinical condition that can be classified into two main types: primary and secondary. Gas enema treatment is usually effective in curing primary intussusception and recurrence is rare [1]. Nevertheless, secondary intussusception may recur and is typically associated with underlying organic pathology. These organic lesions, primarily Meckel’s diverticulum, polyps, tumors, etc., are frequently found at the head of the bowel where secondary intussusception occurs [2, 3].

Small bowel tumors (SBT) are uncommon, especially in children with an incidence of approximately 1 per 100,000. Intussusception caused by SBT is an even rarer condition, and there are fewer instances where pediatric patients specifically seek medical attention for intussusception that is secondary to SBT [4]. As a result, the majority of cases involving intussusception that is secondary to SBT in pediatric patients are typically published as case reports [5–8]. Currently, there is no comprehensive summary available on the epidemiology, clinical features, diagnosis, and clinical outcomes of intussusception that is secondary to SBT in children. In pursuit of this objective, we retrospectively gathered a cohort of pediatric patients with intussusception that was secondary to SBT and treated at our hospital. We meticulously examined their case characteristics and compiled our clinical observations for comprehensive analysis.

## Methods

### Inclusion and exclusion criteria

Patients aged < 18 years with intussusception were enrolled in the current study from January 2016 to January 2022. All patients were diagnosed with intussusception attributable to SBT based on preoperative imaging or surgical findings. Patients were excluded if their clinical data were lost, pathological examination was unclear, or if the intussusception was associated with other types of intestinal polyps. Informed consent was obtained from the parents/guardians of all patients. This study complied with the Helsinki Declaration and was approved by the local ethics committee (approval number: 2022-E-173-R).

### Clinical outcome evaluation indicators

#### Primary outcome

Clinical presentation and pathological findings.

#### Secondary outcomes

Baseline, imaging, surgical and follow-up data.

### Statistical analysis

Statistical analysis was performed using standardized statistical software (Statistical Package for Social Science; version 19.0; SPSS, Inc., Chicago, IL). Non-normally distributed data were expressed as median (25th, 75th interquartile range). Enumeration data were expressed as frequency, rate, or composition ratio, and tested with the chi-squared test. *P* values of < 0.05 were considered statistically significant.

## Results

### **Baseline characteristics & clinical presentation**

This study collected clinical data from 31 children with SBT-associated intussusception, based on the inclusion and exclusion criteria above, with a gender distribution of 24 males and 7 females. The age range of the patients was between 1 month and 11 years and 5 months, with a mean age of 47.4 ± 36.5 months. Among all patients, 26 individuals are over 2 years old, and the distribution of disease onset ages was relatively uniform, with 7 cases in the infant stage, 7 in the toddler stage, 8 in the preschool stage, and 9 in the school-age stage. The duration of illness ranged from 1 to 90 days with a median duration of 5.0 (2.8, 20.3) days.

The major symptoms reported by the patients were abdominal pain, vomiting, and bloody stools. The most commonly reported complaint was recurrent abdominal pain. Other symptoms included recurrent cases of intussusception and wasting. Among all patients, 23 cases (74.2%) presented with abdominal pain as a symptom, mainly characterized by dull pain and inflammatory stimulus-like pain. Thirteen cases (41.9%) exhibited vomiting, while nine cases (29.0%) had bloody stools. Two cases (6.5%) showed signs of wasting (mainly manifested as emaciation and anemia), and postoperative pathology confirmed Burkitt lymphoma and lipoma, respectively. Additionally, two cases (6.5%) of pediatric patients experienced recurrent intussusception, both attributed to Burkitt lymphoma.

### Ancillary examinations

**Fig. 1:**
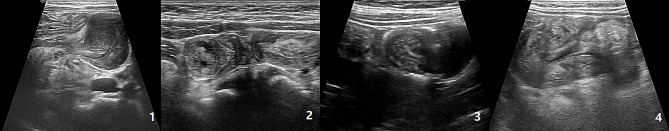
Ultrasound images of SBTs. (1) Lymphoma; (2) Adenomyoma; (3) Inflammatory myofibroblastoma; (4) Lipoma

All patients had preoperative ultrasounds that showed suggestive signs of intussusception, with the typical “concentric circles” pattern observed (Fig. 1). 17 patients had intussusception in the small bowel, while 14 patients had it in the ileocecal region. Out of the 31 patients, 28 suggested that the sleeve’s secondary factor was due to occupation of the intestinal wall. In 8 cases, the tumour and its type were accurately identified, with 5 cases being lymphoma and 3 cases being adenomyoma. Among patients with ileocecal intussusception, only 3 cases showed no evidence of a primary intestinal tumor during preoperative examination.

### Surgical characteristics

**Fig. 2:**
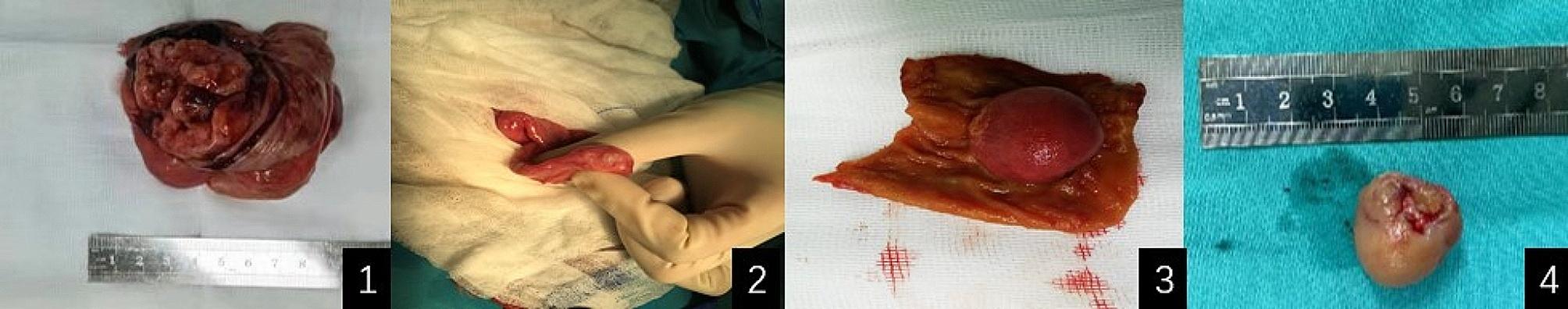
Surgical specimens for SBTs. (1) Lymphoma; (2) Adenomyoma; (3) Inflammatory myofibroblastoma; (4) Lipoma

All surgical procedures went smoothly, and the SBT lesions were visible to the naked eye intraoperatively. The procedures performed included segmental bowel resection with anastomosis in 28 cases and wedge resection of intestinal wall masses (consisted of 2 cases of adenomyoma and 1 case of lipoma). Lymphomas and adenomyomas are more frequently found in the ileum, while myofibroblastomas exclusively occur in the ileum. Only one case of lipoma was detected in the jejunum. Four patients with adenomyoma and one patient with inflammatory myofibroblastoma were found to have experienced intraoperative intestinal necrosis during the course of their surgery. According to our patient information, SBT are more commonly found in the ileum, but there is no significant difference in the prevalence of different types of SBT (Table 1; Fig. 2).

**Table 1:** Pathology and distribution of SBTs [cases]

Distribution	Pathology						
	Lymphoma	Adenomyoma	Inflammatory myofibroblastoma	Lipoma	Total	χ^2^ value	P-value
Jejunum	2	1	0	1	4	-^a^	0.191
Ileum	14	9	3	0	26		
Both	1	0	0	0	1		
Total	17	10	3	1	31	29.400	< 0.001

SBT = Small bowel tumor

^a^Fisher’s exact test, no statistical values

### Pathological characteristics

The pathology results suggested that all 17 cases of lymphoma were indicative of non-Hodgkin’s lymphoma. Among the cases reviewed, there were 9 instances of Burkitt’s lymphoma (average length 19.8 ± 27.0 cm), 5 cases of diffuse large B-cell lymphoma (average length 10.4 ± 5.5 cm), 2 cases of high-grade mature B-lymphocytoma, and 1 case of lymphoblastoma.

### Follow-up & prognosis

All patients were followed up for a period of 3 months to 6 years. Only one patient with adenomyoma experienced a recurrence of primary intussusception one week after surgery. The remaining patients did not have any surgery-related complications in the short or long term. Patients with lymphoma were monitored for a period ranging from 6 m to 6y. Among the 11 children who were monitored for 6 m to 5y, none had a recurrence of the condition. Four patients were lost to follow-up, and four died. Patients with inflammatory myofibroblastoma, adenomyoma, and lipoma were monitored for a period ranging from 6 months to 5.5 years. In patients with adenomyoma and lipoma who underwent wedge resection of the intestinal wall mass, the prognosis was not bad as there were no reported cases of recurrence or metastases in the digestive tract or elsewhere during the follow-up period.

## Discussion

The small intestine comprises roughly 75% of the total length and 90% of the total surface area of the gastrointestinal tract (GI) [4]. Tumors of the small intestine are considered rare, with an incidence rate of 0.01 per 1,000 population [4]. The occurrence of SBT in children is even more infrequent. Moreover, the clinical manifestation of SBT in children is nonspecific, and recurrent intussusception is the most common initial presentation. Intussusception is more common in children, with 80% of cases occurring in patients aged ≤ 2 years, and primary intussusception is the most common form [9–11]. However, as in our study, where 26 patients were above the age of 2 years, it is consistent with the fact that secondary intussusception is more prevalent in older children. In many cases, emergency surgery is necessary for these patients, and a definitive diagnosis typically requires pathological examination of the specimen.

There are often distinguishing features between the two types of intussusceptions, which can aid the doctor in making a differential diagnosis. These features include clinical symptoms, disease history and supplementary tests. The classic clinical symptoms of primary intussusception are characterized by the triad of intermittent abdominal pain, vomiting, and bloody stools [12]. In contrast, secondary intestinal obstruction usually lacks the simultaneous presence of all three typical symptoms [13]. Out of our patients, only three exhibited the classic triad of abdominal pain, vomiting, and bloody stools, while the majority presented with recurrent abdominal pain. The disease duration of primary intussusception is usually less than 1d. But if the disease persists for longer than 3 days, physicians should be vigilant in detecting potential triggers [14]. The extended duration of illness in the majority of our patients, with 28 cases (82.4%) persisting for more than 3 days, corroborated their view. However, the above evidence is insufficient to support a diagnosis of intussusception and SBT. Intussusception is typically diagnosed with ultrasound alone, but only 61.4% of positive cases clearly indicate the presence of an inciting factor [15]. Based on the ultrasound findings, all patients show evidence of intussusception and are able to differentiate the type. Out of the patients included in our study, 28 cases indicated the presence of secondary factors, while only 8 suggested the presence of SBT. Among the 31 patients with intussusception, 3 cases (10.0%) of ileocolic intussusception showed no indications of secondary factors on ultrasound examination. Upon reviewing their medical records, it was discovered that the tumors measured 0.5cm1.0 cm (adenomyoma with concurrent intestinal necrosis), 1.8 cm*1.1 cm (adenomyoma with concurrent intestinal necrosis), and 0.5 cm*0.7 cm (lymphoblastic lymphoma without any signs of intestinal necrosis). Considering these factors, we believe that the small size of the tumor and the potential presence of intestinal necrosis may increase the difficulty of preoperative ultrasound diagnosis. While a definitive preoperative diagnosis in children with intestinal intussusception caused by SBT may present challenges, we recommend, drawing from our clinical expertise, that SBT should be considered in cases where children have a history of intestinal intussusception lasting over 3 days and are aged above 2 years. In addition, if ultrasound indicates the presence of secondary factors, caution should be raised regarding the possibility of SBT.

Currently, more than 40 distinct histological types of benign and malignant neoplasms are recognized as solid pseudopapillary tumors (SBTs). Among benign lesions in adults, common types include leiomyoma, adenoma, lipoma, and hamartoma. Malignant lesions often comprise adenocarcinoma, neuroendocrine tumors, sarcoma, and lymphoma, as highlighted in references [16–19]. However, the pathological spectrum of SBTs in children differs significantly from that observed in adults. In our study, lymphoma constituted 55.9% of all tumors, while adenomyoma, inflammatory myofibroblastic tumor, and lipoma accounted for 29.4%, 11.8%, and 2.9%, respectively.

While intussusception in children traditionally occurs in the ileocolic region, our findings suggest that secondary intussusception resulting from small intestinal tumors in children is more likely to manifest as small bowel intussusception. This observation aligns with previous research by Lin et al. [20], who identified small bowel intussusception as the predominant type in secondary intussusception cases.

The primary treatment approach for intussusception in children secondary to SBT involves surgical intervention, with segmental resection and wedge resection being the principal surgical modalities [21]. Although postoperative pathology is crucial for a definitive diagnosis of SBT, our experience indicates that intraoperative assessment of tumor morphology can aid in identifying its pathological type. Lymphoma and inflammatory myofibroblastoma typically present with distinctive surface characteristics, such as surface indentations on the plasma membrane and rounded, granular, or lobulated protrusions on the mucosal surface, with a firm texture. However, distinguishing between these two types can be challenging. Therefore, in cases involving either type of tumor, consideration should be given to performing a segmental bowel resection. Gross visualization during surgery is usually sufficient to ensure complete removal of the tumor [4]. Although intraoperative frozen section pathology was not performed in our cases, post-operative pathology confirmed complete tumor removal.

Lymphoma can manifest in various locations, necessitating a thorough examination of the entire small bowel. The serosal surface of adenomyoma exhibits bubble-like protrusions, while the mucosal surface appears as smooth protrusions. Lipomas, on the other hand, present as polypoid lesions protruding from the mucosal surface, occasionally with ulceration at the base, making them challenging to distinguish from polyps. Nevertheless, based on their gross morphology, adenomyomas and lipomas can be identified as benign tumors. Depending on their size, segmental intestinal resection or wedge resection may be performed [22].

Subsequently, the prognosis of SBT in children is contingent upon its pathological type. Chemotherapy emerges as the primary treatment option for gastrointestinal lymphoma, while surgery is generally reserved for patients facing challenging diagnoses, gastrointestinal perforations, and intestinal obstruction [23, 24]. Complete resection remains the principal treatment modality for other tumor types, with incomplete resection standing as a significant contributor to recurrence [25].

## Conclusions

Intussusception secondary to SBT in pediatric patients represents a rare and challenging condition for preoperative diagnosis. Surgical intervention is essential for children with recurrent episodes of intussusception and for those in whom ultrasound indicates the presence of a “primary cause,” with the definitive diagnosis often dependent on intraoperative observations and postoperative pathology. This study compiles the clinical characteristics of pediatric patients diagnosed with SBT in our hospital over recent years, thereby furnishing valuable clinical insights for both pediatric emergency surgery and general surgical practitioners.

## Data Availability

The datasets used and/or analysed during the current study are available from the corresponding author on reasonable request.
